# Long Non-coding RNA MALAT1: A Key Player in Liver Diseases

**DOI:** 10.3389/fmed.2021.734643

**Published:** 2022-01-25

**Authors:** Juan Lu, Jing Guo, Jun Liu, Xiaomin Mao, Kaijin Xu

**Affiliations:** ^1^State Key Laboratory for Diagnosis and Treatment of Infectious Diseases, National Clinical Research Center for Infectious Diseases, Collaborative Innovation Center for Diagnosis and Treatment of Infectious Diseases, College of Medicine, The First Affiliated Hospital, Zhejiang University, Hangzhou, China; ^2^Haining People' Hospital, Haining Branch, The First Affiliated Hospital, College of Medicine, Zhejiang University, Haining, China

**Keywords:** lncRNAs, MALAT1, mechanism, signaling pathways, liver diseases

## Abstract

Long non-coding RNAs (lncRNAs) exceed 200 nucleotides in length are considered to be involved in both developmental processes and various diseases. Here, we focus on lncRNA MALAT1 (metastasis-associated lung adenocarcinoma transcript 1), which was one of the most important lncRNAs in proliferation, apoptosis, and migration. MALAT1 plays a regulatory role in liver diseases, including hepatic fibrosis, liver regeneration, liver cancer, and fatty liver diseases. In the current review, we summarize the latest literature about the function roles of MALAT1 in liver disorders. Probing the regulatory mechanism and cross talk of MALAT1 with other signaling pathways of pathological processes would improve the prognosis, diagnosis of liver diseases, and offer a promising candidate target for therapeutic interventions.

Seventy-five percent of the human genome generates transcripts, yet only about two percent of the genome encodes proteins. The majority of transcripts, including microRNAs (miRNAs) and long non-coding RNAs (lncRNAs), do not encode proteins (1). Small non-coding RNAs of ~22 nucleotides regulate the translation and stability of mRNA at the post-transcriptional level. Several biological processes are regulated by lncRNAs (more than 200 nucleotides), including carcinogenesis, development, and differentiation (2, 3). lncRNAs modulate various cellular processes, including nuclear organization and transcriptional and post-transcriptional modulation of gene expression (4, 5). lncRNAs serve as competitive endogenous RNAs (ceRNAs) in a regulatory network by “sponging” target miRNAs to regulate mRNA expression. This regulatory network is implicated in cancer development, apoptosis, and drug resistance (6–8).

To date, over 50,000 human lncRNAs have been identified (9). One of the most extensively studied of lncRNAs is Metastasis-associated lung adenocarcinoma transcript 1 (MALAT1, ENSG00000251562), located in human chromosome 11q13.1 (mouse chromosome 19qA); MALAT1 is also known as nuclear-enriched abundant transcript first described to be associated with non-small cell lung cancer (10–12). Elevated MALAT1 expression is implicated in hyperproliferation, cellular and molecular functions, metastasis, and poor prognosis (13, 14). MALAT1 is overexpressed in a variety of human diseases in the form of a ceRNA network, which is important for gene expression, proliferation, and metastasis (15). The pathological processes would be activated once the level of MALAT1 expression changes to abnormal condition due to various endogenous or exogenous inducers. Here, we summarize the roles of MALAT1 in liver diseases including hepatic fibrosis, hepatic carcinoma, liver regeneration, and fatty liver diseases.

## Molecular Function of MALAT1

MALAT1 is preferentially associated with transcriptionally active genes in the nucleus (16). MALAT1 plays a very critical cellular function for normal physiology. Given the nuclear localization of nuclear speckles, MALAT1 could be implicated in the regulation of alternative splicing in terms of binding splicing factors (17). The interaction between MALAT1 and the splicing factors of serine/arginine-rich (SR) proteins regulates selective splicing by modulating phosphorylation and distribution in the nuclear macular region. MALAT1 also regulates SR proteins by affecting the localization and activities of shear factor kinases such as serine/arginine protein kinases 1 (SRPK1) (18, 19). As a result of MALAT1 transcripts located in the nucleus, MALAT1 combined with release of pre-mRNA splicing factors via antisense oligonucleotides triggers could induce overexpression of the related factors. Therefore, MALAT1 might serve as an “anchor point” for localization of certain genes near nuclear speckles and induce transcriptional activation to affect the processing of their RNAs, implicating MALAT1 in transcriptional regulation (20). The major mechanisms of post-transcriptional regulation of MALAT1 include alternative splicing, protein activities, and competitive ceRNAs (15). MALAT1 is also expressed in vascular endothelial cells and plays important roles in regulating vascular growth. The cell cycle inhibitory proteins were also significantly increased accompanied by the depletion of MALAT1 (21).

**Figure 1:**
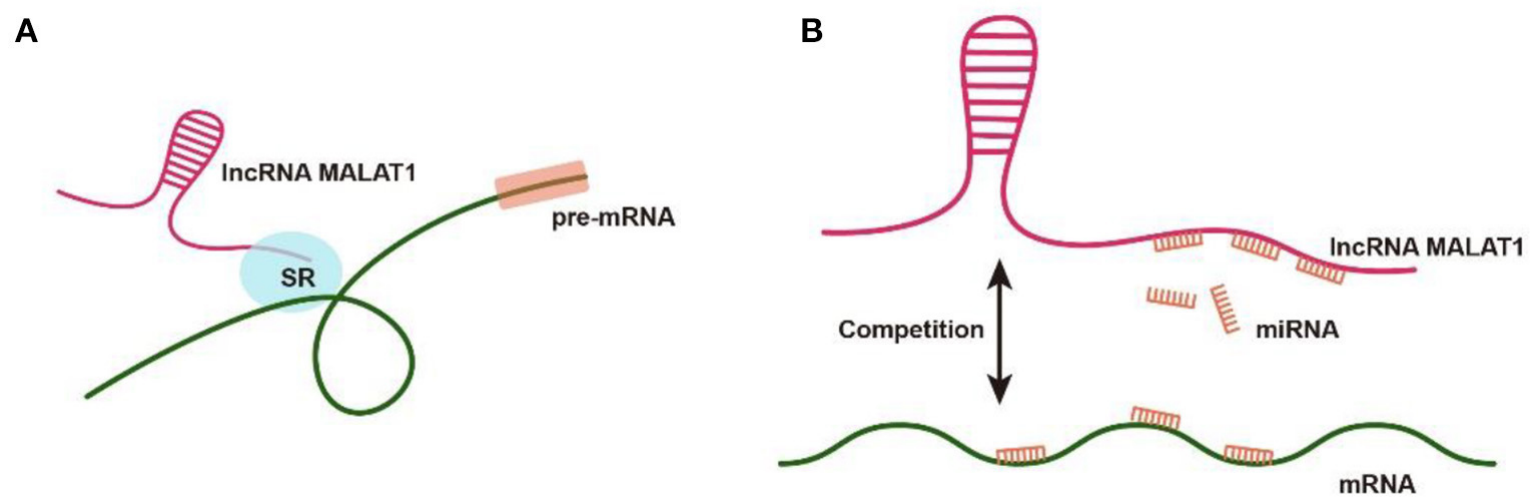
Molecular Function of MALAT1. **(A)** The transcriptional regulation of MALAT1 by interaction between MALAT1 and SR splicing factors. **(B)** The post-transcriptional regulation of MALAT1 via competitive ceRNAs.

MicroRNAs bind to the 3'UTR of their target genes and negatively regulate gene expression through depressing translation or promoting mRNA attenuation. This strategy was proposed initially by Poliseno to explain how mRNAs communicate with lncRNAs through miRNA response elements (MREs) as “language” (22). In the process of RNA-miRNA regulation, MREs act as binding sites for ceRNAs, which positively adjust miRNAs availability to bind their target mRNAs (23). MALAT1 also followed the ceRNA regulatory system through the negatively regulation between MALAT1 and micRNAs (Figure 1).

MALAT1 binds to chromatin of activated transcribing genes, transcription factors and transcriptional co-activators and regulates their expression at the transcriptional level to promote cell proliferation and inhibit cell apoptosis. MALAT1 was also triggered to upregulate transcriptional activators of proteosome genes (24).

## MALAT1 –an Inducer in Hepatic Fibrosis

Hepatic fibrosis is characterized by chronic abnormal hyperplasia and accumulation of large amounts of extracellular matrix (ECM) [including smooth muscle actin (SMA) and type I collagen], and the release of proinflammatory and profibrotic factors. Fibrosis can result from a variety of chronic liver diseases such as viral hepatitis, alcoholism, drug abuse, metabolic syndrome, genetic metabolic diseases, and autoimmune hepatitis (25). The regulatory mechanism of liver fibrosis is not entirely clear. The activation of hepatic stellate cells (HSCs), the resident perisinusoidal cell type, is critical in the development of liver fibrosis (26). Activated HSCs are considered proliferative cells that secrete profibrogenic mediators and express ECM; they may also be involved in fibrosis progression (27). Yu et al. (28) found that MALAT1 was significantly upregulated in activated HSCs and negatively correlated with the expression of miR-101b in in the mouse liver fibrosis model induced by carbon tetrachloride (CCl4). MALAT1 and ras-related C3 botulinum toxin substrate 1 (Rac1) are targets of miR-101b, and the former acts as a ceRNA to enhance the expression of Rac1, thus promoting HSC proliferation and activation. Dai et al. (29) found that MALAT1 extracted from arsenite-treated human hepatocytes promoted the activation of LX-2 HSCs (an immortalized HSC cell lines) by binding miRNA-26b. Wu et al. (30) reported that MALAT1 influenced the progression of hepatic fibrosis by repressing the expression and function of silent information regulator 1 (SIRT1), a nicotinamide adenine nicotinamide (NAD+)-dependent III class of histone deacetylases in the Sirtuin family (16). SIRT1 suppressed the ability of the promoters of fibrogenic genes, such as collagen type I, to bind Smad3, a downstream mediator of the TGF-β signaling pathway (31, 32). In short, MALAT1 promotes HSC activation by blocking SIRT1-mediated inhibition of the TGF-β signaling pathway in hepatic fibrosis.

## MALAT1—a Regulating Role in Liver Regeneration

The normal liver has a strong regenerative ability and can maintain the original liver volume by inducing mitosis of its own cells, or tissue repair through rapid division and proliferation from endogenous liver stem cells (such as liver oval cells) to mature liver cells (33). Because of the limited regenerative ability of endogenous liver stem cells, exogenous measures are needed to promote liver regeneration and maintain the liver function of patients with severe liver injury or liver failure (34). Liver cells begin proliferate and divide in the initial period. The proliferation of liver is not only completed by parenchyma cells, but by a variety of cells which also participate in the coordination and completion at the stage of proliferation (35). The timely and automatic termination of hepatocyte proliferation is distinct from the unlimited proliferation of liver tumors in the termination stage, which are are important for the liver regulation of growth, development, and differentiation (36). The interaction and regulatory factors were activated rested during the biological processes of liver development, liver regeneration and hepatic carcinoma. Tripathi et al. (37) showed that MALAT1 promotes the proliferation of normal cells. MALAT1 also promotes the expression of cell cycle genes (such as the transcription factor B-myb) and progression from G1 to S phase, thus inducing mitosis. Knockout of MALAT1 activated p53 and its reporter gene, a downstream gene of MALAT1. Maxy et al. (38, 39) studied the expression of MALAT1 in various tissues and organs of mice and found that MALAT1 expression in mice was highest in the liver followed by the kidney, lowest in skeletal muscle. MALAT1 expression was elevated, and its inhibition or knockout may promote the proliferation, migration, and tubulation, and suppress apoptosis, of endothelial cells by activating the PI3K/Akt signaling pathway (40).

Liver regeneration is initiated immediately after liver resection, and upregulation of hepatocyte growth factor (HGF) induces the expression of MALAT1, damaging the stability of the p-catenin degradation complex (41). The resulting increased total p-catenin activates the Wnt/β-catenin signaling pathway and upregulates cyclinD1, promoting progression from G1 to S phase and shortening the cell cycle, thereby accelerating liver cell proliferation and liver regeneration (42). Deficiency of MALAT1 inhibits VEGFR2 expression, reduces angiogenesis, perfusion, and functional recovery of ischemic hind limbs in mice, and inhibits blood flow recovery and capillary density in gastrocnemeal muscle tissue after ischemia, suggesting that MALAT1 affects angiogenesis via multiple mechanisms (21). MALAT1 regulates angiogenesis and immune responses (43). MiR-3064-5p inhibits the FOXA1/CD24/Src pathway to exert an antiangiogenic effect, whereas MALAT1 adsorbs miR-3064-5p, thereby alleviating the inhibition of FOXA1 and promoting hepatic hemangiogenesis (44). Hou et al. (45, 46) confirmed that MALAT1 is an important upstream target of miR-140, which inhibits VEGF-A expression and M2 macrophage polarization, thereby inhibiting angiogenesis and immunosuppression.

## MALAT1—a New Therapeutic Target in Hepatic Carcinoma

The expression of MALAT1 is significantly increased in HCC tissues and cell lines, which promotes HCC proliferation and metastasis and inhibits HCC cell apoptosis by acting as an oncogene (47–49). Silencing of MALAT1 reduces the proliferation, invasion, and migration of cancer cells, and induces their apoptosis (50). The upstream of MALAT1 contains 5 specific protein 1/3(SP1/3) binding sites, and the combined regulation of SP1 and SP3 in cancer cells promote the expression of MALAT1. Yes-related protein (YAP) upregulates the expression of MALAT1 at the transcriptional and post-transcriptional levels, whereas serine/arginine splicing factor 1 (SRSF1) rich in serine/arginine had the opposite effect. Overexpression of YAP reduces SRSF1 nuclear retention (51).

MALAT1 plays a role in HCC cell proliferation and apoptosis by multiple pathways. Upregulation of MALAT1 promotes cancer cell proliferation, and its downregulation promotes cancer cell death and autophagy. Malakar et al. (52) found that high expression of MALAT1 in HCC cells upregulates oncogenic shear factor SRSF1 and activates the mTOR pathway, thereby promoting the proliferation and survival of HCC cells. Peng et al. (50) found that MALAT1 expression regulates the proliferation, apoptosis, and autophagy of HCC cells by adsorption of miR-146a, whereas downregulation of miR-146a upregulates PI3K, modulating the phosphorylation of downstream Akt and mTOR. Therefore, apoptosis and autophagy of HCC cells can be inhibited by targeting the PI3K/Akt/mTOR signaling axis. Liu et al. (53) showed that MALAT1 acted as a molecular sponge to absorb miR-195, which inhibited its downstream target EGFR, inducing the activation of PI3K/Akt and JAK/STAT pathways by overexpression of EGFR, thus promote the growth activity of HCC cells. Chen et al. (54) reported that MALAT1 regulates the expression of zinc finger E-box binding homeobox (ZEB1) by sponging miR-143-3p. In conclusion, the up- or downregulation of MALAT1 is related to the proliferation and apoptosis of HCC cells. MALAT1 acts as a molecular sponge to regulate miRNA signaling pathways affecting downstream factors and promotes HCC cell proliferation and inhibits their apoptosis.

HCC invasion and metastasis are aggravated via signaling pathways and MALAT1. YAP1 in vascular endothelial cells reduces vascular proliferation (55). Exosomes containing MALAT1 are released into the tumor microenvironment, inhibiting and depleting YAP1, activating ERK1/2 signal transduction, and enhancing the expression of MMP2 and MMP9, thereby promoting tumor invasion and metastasis. The tumor transcription factor FOXM1 is the target of miR-125a-3p, targeting of which by MALAT1 upregulates FOXM1 expression and promotes HCC invasion and migration (51, 56). SNAI1, a key transcription factor in the epithelial–mesenchymal transition, is also a direct target of miR-22, which could be absorbed by MALAT1, promoting the enrichment of enhancer of zeste homolog 2 (EZH2) to inhibit miR-22 transcription, thereby upregulating SNAI1 expression and facilitating HCC invasion and distant metastasis. Li et al. (57) reported that MALAT1 acted as a molecular sponge for miR-146b-5p, inhibiting HCC growth and metastasis by targeting Akt phosphorylation mediated by TNF receptor-related factor 6. Hou et al. (58) found that MALAT1 reduced the inhibition of SIRT1 by miR-204 by competitively binding miR-204. Chen et al. (54) showed that MALAT1 regulates ZEB1 expression by sponging miR-143-3p, promoting HCC invasion and metastasis.

MALAT1 also promotes glycolysis in HCC. Aerobic glycolysis (Warburg effect) is a marker of tumor cells' ability to escape apoptosis to promote proliferation and migration. It could provide material basis for tumor cells and create an acidic microenvironment (59, 60). Malakar et al. (61) found that MALAT1 enhances the translation of metabolic transcription factor TCF7L2 by upregulating shear factor SRSF1 and activating the mTORC1-4EBP1 axis to upregulate glycolysis genes and inhibit gluconeogenesis, promoting the development of HCC (62). Additionally, it has been shown that MALAT1 enhances glycolysis in liver, and inhibits gluconeogenesis, via elevated translation of the transcription factor TCF7L2 and as such also plays a crucial role in metabolic stress (63). MALAT1 promotes the progression of inflammation-associated HCC. Huang et al. (64) reported that MALAT1 induces the secretion of inflammatory cytokines, promoting the progression of inflammation-related HCC by mobilizing chromatin and remodeling subunit brahma-related gene 1 (BRG1) to the promoter region of the inflammatory cytokines IL-6 and C-X-C motif chemokine ligand 8 (CXCL8).

Cancer stem cells (CSCs) are characterized by self-renewal and differentiation and are considered the seeds of tumor genesis, development, and metastasis. MALAT1 promotes HCC stem cell properties. The higher the proportion of CSCs, the more aggressive the tumor (65). He et al. (4) reported that HBx protein induced CSC production in HCC via the PI3K/Akt signaling pathway (66). MALAT1 emerged as the function of competing endogenous RNA, preventing miR-124-mediated inhibition of PI3K/Akt signaling. This induced CSC characteristics and ultimately promoted HBV-associated HCC. Chen et al. (67) reported that circ-MALAT1 generated by reverse shear of lncRNA MALAT1 acts as a “brake” in the ribosome, and forms a complex with the ribosome and mRNA, preventing the transcription factor PAX5 mRNA translation and promoting CSC self-renewal. However, circ-MALAT1 also acts as a sponge for miR-6887-3p to enhance the phosphorylation of JAK2, activating the JAK2/STAT3 signaling pathway and promoting CSC self-renewal (68). Thus, MALAT1 represents both a promising cancer bio-marker as well as a potential therapeutic target for limiting metastatic growth.

## MALAT1 in Other Liver Diseases

**Figure 2:**
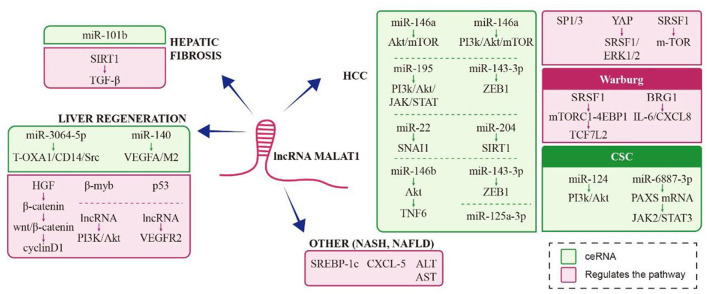
Schematic representation of MALAT1 in liver diseases.

MALAT1 significantly inhibits palmitic acid-induced lipid accumulation and increases expression of SREBP-1c, an important regulator of cholesterol and fatty acid synthesis in abnormal lipid metabolism and fatty liver disease (69, 70). MALAT1 abundance in liver tissue is closely related to the pathological changes in non-alcoholic fatty liver disease (NAFLD), a clinicopathologic syndrome characterized by diffuse bullae steatosis (abnormal accumulation of lipids in liver tissues) not caused by alcohol or other hepatotoxic factors. MALAT1 may mediate chemokines by regulating C-X-C motif chemokine ligand 5 (CXCL5) in hepatic stellate cells in the occurrence of non-alcoholic steatohepatitis (NASH) and fibrosis in patients with NAFLD (71, 72). MALAT1 abundance increases significantly in NASH with hepatocyte ballooning degeneration and lobular inflammation, and in hepatocyte dysfunction with elevated alanine aminotransferase, aspartate aminotransferase, and alkaline phosphatase. The schematic representation of MALAT1 in liver diseases was Figure 2. A form was also presented to explain different roles of MALAT1 under different liver diseases and how MALAT1 influences pathophysiology in Table 1.

**Table 1:** Different roles of MALAT1 under different liver diseases.

**Hepatic fibrosis**	**Promoting proliferation by ceRNA of miR-101b**
Liver regeneration	Promoting proliferation by ceRNA of miR-3064-5p140
Hepatic carcinoma	Promoting proliferation by ceRNA of miR-146a/195/143-3p/ 22/204/146b/125a-3p
	Promoting proliferation by upregulating SP1/3/YAP/SRSF1
	Promoting proliferation by Warburg effect
	Promoting proliferation by ceRNA of miR-124/6887-3p
Fatty liver disease	Promoting proliferation by upregulating SREBP-1c/CXCL5/ transaminase

## Conclusion

LncRNA is a hot research topic in the field of liver disease in recent years. As a member of the lncRNA family, MALAT1 is a multi-functional lncRNA and an important regulator in hepatic fibrosis, liver regeneration, cancer, and fatty liver diseases. The molecular mechanisms mediated by one lncRNA would be complicated. Compared to most lncRNAs, MALAT1 is expressed at relatively high level in almost all human tissues in a variety of regulating pathways, thus makes it intricate to be targeted by simply silencing or overexpressing in pathological conditions. Further challenge to therapeutically measures would include small molecules specifically designed to intervene the gene-protein interaction for MALAT1 functions (73). A deeper understanding of the functions of MALAT1 and its interaction network will lay the foundation for the development of lncRNAs as therapeutic targets and as diagnostic or prognostic biomarkers for liver diseases.

## Author Contributions

JLu and KX structured the text and content. JLu and JG wrote and edited the manuscript. JLi generated the figures. XM reviewed the literature. All authors contributed to the article and approved the submitted version.

## Funding

This research was funded by the Independent Project Fund of the State Key Laboratory for Diagnosis and Treatment of Infectious Diseases, the National Key Research and Development Program of China (No. 2016YFC1101404/3), and Zhejiang Basic Public Welfare Research Program of China (No. LQ20H030012).

## Conflict of Interest

The authors declare that the research was conducted in the absence of any commercial or financial relationships that could be construed as a potential conflict of interest.

## Publisher's Note

All claims expressed in this article are solely those of the authors and do not necessarily represent those of their affiliated organizations, or those of the publisher, the editors and the reviewers. Any product that may be evaluated in this article, or claim that may be made by its manufacturer, is not guaranteed or endorsed by the publisher.

## Figures and Tables

**Table:** 

**General**	**Abbreviation full name**
4EBP1	phosphorylated 4E-binding protein 1
AKP	alkaline phosphorus
ALT	Aminotransferase
AST	aspartate aminotransferase
BRG1	brahma-related gene 1
CCl4	carbon tetrachloride
ceRNAs	competitive endogenous RNAs
CSC	cancer stem cells
CXCL5	C-X-C motif chemokine ligand 5
CXCL8	C-X-C motif chemokine ligand 8
ECM	extracellular matrix
EZH2	enhancer of zeste homolog 2
FOXA1	forkhead box A1
HCC	hepatic carcinoma
HGF	hepatocyte growth factor
HSCs	hepatic stellate cells
lncRNA	long noncoding RNA
MALAT1	metastasis-associated lung adenocarcinoma transcript 1
miRNA	microRNA
MREs	microRNA response elements
mTORC1	mechanistic target of rapamycin complex 1
NAD+	nicotinamide adenine dinucleotide
NAFLD	non-alcoholic fatty liver disease
NASH	nonalcoholic steatohepatitis
NEAT2	uuclear-enriched abundant transcript 2
Rac1	ras-related C3 botulinum toxin substrate 1
SIRT1	silent information regulator 1
SMA	smooth muscle actin
SMA	smooth muscle actin
SNAI1	snail family transcriptional repressor 1
SP1/3	specific protein 1/3
SR proteins	serine/arginine-rich proteins
SRPK1	serine/arginine protein kinases 1
SRSF1	serine/arginine splicing factor 1
STAT	signal transducer and activator of transcription
TCF7L2	transcription factor 7-like 2
TNF	tumor necrosis factor
YAP	Yes-related protein
ZEB1	zinc finger E-box binding homeobox 1

**Table:** 

**Signaling pathway**	**KEGG ID**
AKT/mTOR signaling pathway	hsa04150
CXCL5 signaling pathway	hsa04060
CXCL8 signaling pathway	hsa04061
cyclinD1 signaling pathway	hsa05200
EFGR signaling pathway	hsa01521
ERK1/2 signaling pathway	hsa04933
FOXM1 signaling pathway	hsa04218
IL-6 signaling pathway	hsa05200
JAK/STAT signaling pathway	hsa04630
JAK/STAT signaling pathway	hsa04630
MAPK1 signaling pathway	hsa05166
MMP2/9 signaling pathway	hsa05219
mTOR1-4EBP1signaling pathway	hsa04218
P53 signaling pathway	hsa04115
PAX5 signaling pathway	hsa05202
PI3K/AKT signaling pathway	hsa04151
SIRT1 signaling pathway	hsa04211
SNAI1 signaling pathway	hsa04520
Src signaling pathway	hsa04510
TCF7L2 signaling pathway	hsa05200
TGF-β signaling pathway	hsa04068
VEGF signaling pathway	hsa04210
Wnt/b-catenin signaling pathway	hsa04310
YAP/SRSF signaling pathway	hsa04390
ZEB1 signaling pathway	hsa05206

**Table:** 

**Mirbase ID**	**HGNC symbol**	**Mirbase accession**
has-miR-124	MIR124	MI0000443
has-miR-143-3p	MIR143	MI0000459
has-miR-146a	MIR146A	MI0000477
has-miR-146b	MIR146B	MI0003129
has-miR-22	MIR22	MI0000078
has-miR-3064-5p	MIR3064	MI0017375
hsa-miR-125a	MIR125A	MI0000469
hsa-miR-140	MIR140	MI0000456
hsa-miR-195	MIR195	MI0000489
hsa-miR-204	MIR204	MI0000284

**Table:** 

**HGNC symbol**	**Full name accession**	**Accession ID**
4EBP1	phosphorylated 4E-binding protein 1	ENSG00000258870
AKT	serine/threonine kinase 1	ENSG00000142208
β-catenin	β-catenin	ENSG00000168036
BRG1	brahma-related gene 1	ENST00000644760
CXCL5	C-X-C motif chemokine ligand 5	ENSG00000163735
CXCL8	C-X-C motif chemokine ligand 8	ENSG00000169429
EGFR	growth factor receptor	ENSG00000146648
ERK1	extracellular signal-regulated kinase 1	ENSCING00000007067
EZH2	enhancer of zeste homolog 2	ENSG00000106462
FOXA1	forkhead box A1	ENSG00000129514
IL6	interleukin 6	ENSG00000136244
JAK2	janus kinase 2	ENSG00000096968
MALAT1	metastasis-associated lung adenocarcinoma transcript 1	ENSG00000251562
MAPK	mitogen-activated protein kinase	ENSG00000100030
MMP2	matrix metallopeptidase 2	ENSG00000087245
MMP-9	matrix metallopeptidase 9	ENSG00000100985
MTOR	mechanistic target of rapamycin kinase	ENSG00000198793
PI3K	phosphatidylinositol-4,5-bisphosphate 3-kinase	ENSG00000121879
SIRT1	silent information regulator 1	ENSG00000096717
SNAI1	snail family transcriptional repressor 1	ENSG00000124216
SRPK1	serine/arginine protein kinases 1	ENSG00000096063
TCF7L2	transcription factor 7-like 2	ENSG00000148737
TGF-β	transforming growth factor β	ENSG00000105329
VEGFR2	vascular endothelial growth factor receptor 2	ENSG00000112715
WET2	wnt family member 2	ENSG00000105989
ZEB1	zinc finger E-box binding homeobox 1	ENSG00000148516

## Glossary

### Abbreviations

The following abbreviations are used in this manuscript:
